# Bilateral Spontaneous Pneumothorax in a Young Gentleman

**DOI:** 10.1002/rcr2.70347

**Published:** 2025-09-29

**Authors:** Albert Teng, Tzy Harn Chua, Chun Yuen Chow, Yi Hern Tan

**Affiliations:** ^1^ Department of Respiratory and Critical Care Medicine Singapore General Hospital Singapore Singapore; ^2^ Department of Anatomical Pathology Singapore General Hospital Singapore Singapore

**Keywords:** bilateral spontaneous pneumothorax, cystic lung disease, pulmonary cysts, pulmonary Langerhans cell histiocytosis, secondary

## Abstract

Bilateral spontaneous pneumothorax is a rare condition that occurs mainly in patients with underlying lung disease. We report the case of a 26‐year‐old Chinese male with a history of smoking who presented with sudden onset of chest tightness and dyspnoea. Chest radiography revealed bilateral pneumothoraxes, prompting the insertion of bilateral chest tubes. Further imaging with high‐resolution computed tomography of the chest revealed multiple bilateral, thin‐walled cysts with relative sparing of the lung bases and tiny peripheral nodules. The patient underwent bilateral video‐assisted thoracoscopic surgery with bullectomy, pleurodesis, and surgical lung biopsy. Histopathological examination revealed cysts lined by Langerhans cells with eosinophilic infiltration, establishing the diagnosis of pulmonary Langerhans cell histiocytosis (PLCH). This case highlights the importance of considering cystic lung diseases, particularly PLCH, in young smokers presenting with spontaneous bilateral pneumothorax. Radiologic and histopathologic correlation is essential for definitive diagnosis. Smoking cessation remains the cornerstone of management.

## Graphical abstract

We present a case of bilateral spontaneous pneumothorax in a 26‐year‐old Chinese male with a history of smoking. Imaging reveals the presence of underlying cystic lung disease and he subsequently underwent bilateral video‐assisted thoracoscopic surgery (VATS) bullectomy, mechanical pleurodesis, and surgical lung biopsy. This case highlights the importance of considering secondary causes, in particular cystic lung diseases, in patients presenting with spontaneous bilateral pneumothorax.

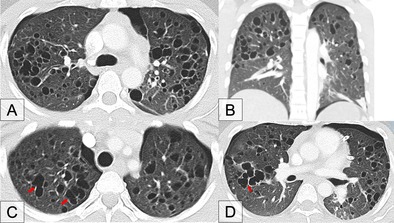

## 1 Introduction

Pneumothorax refers to the presence of air in the pleural cavity, leading to partial or complete lung collapse. Bilateral spontaneous pneumothorax is a rare clinical entity, seen in only about 1.3% of spontaneous pneumothorax cases [1]. It is typically associated with underlying lung pathology. We describe a young patient presenting with bilateral pneumothorax.

## 2 Case Report

A 26‐year‐old previously well Chinese gentleman presented with sudden onset shortness of breath and chest tightness.

On arrival to the emergency department (ED), he was afebrile, with a blood pressure of 122/73 mmHg, heart rate of 116 and oxygen saturation of 100% on oxygen supplementation of 50% oxygen via a venturi mask. It was noted that his oxygen saturations in the ambulance were around 80% on room air. Chest examination revealed reduced air entry bilaterally with no wheeze or crepitations appreciated.

**FIGURE 1:**
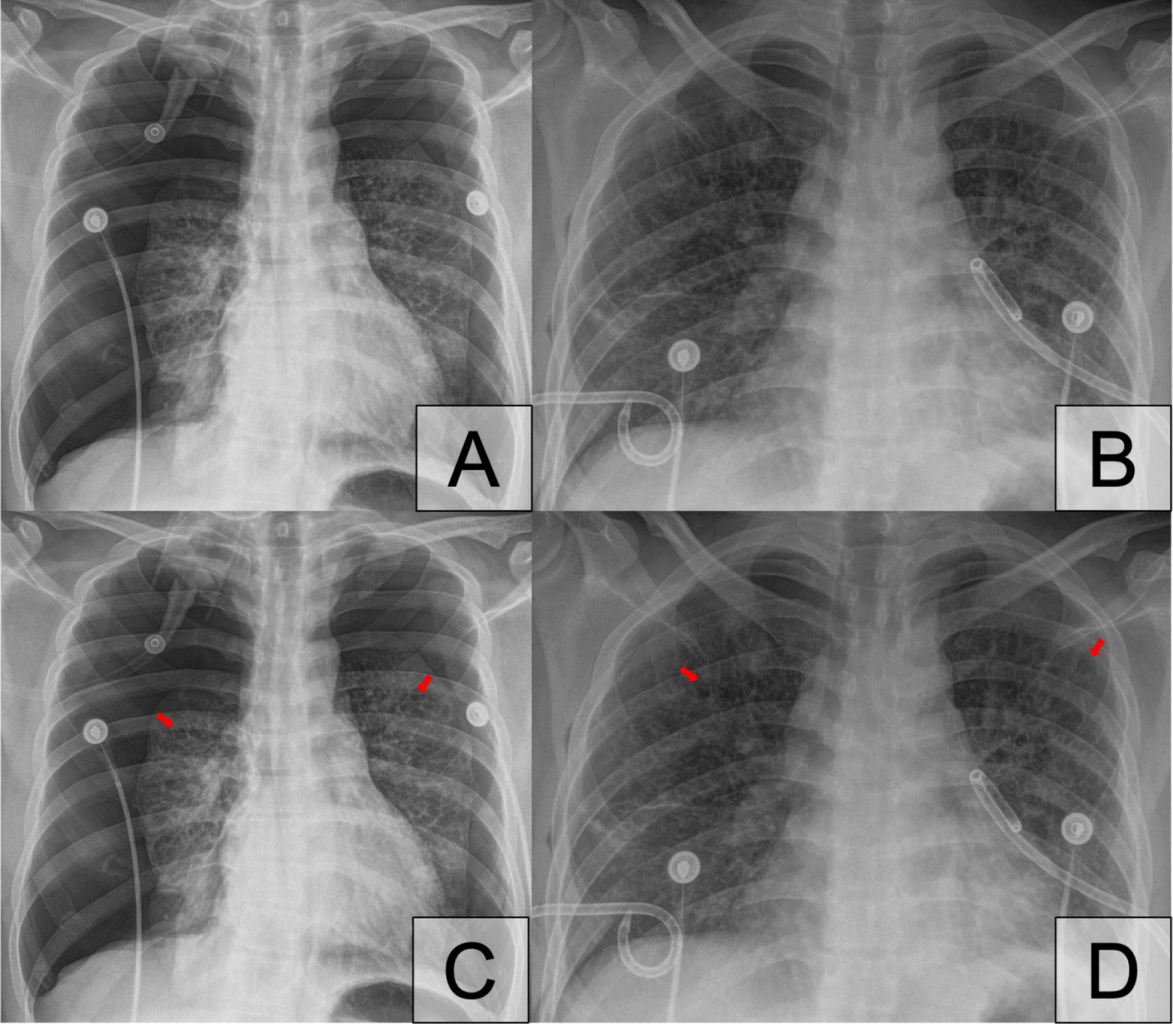
A: Chest radiograph on arrival to Emergency Department. B: Chest radiograph after insertion of bilateral chest tubes. C and D: Arrows indicating airspace lucencies.

Chest radiograph showed bilateral pneumothorax (Figure 1A).

Bilateral 14 Fr chest tubes were inserted and repeat chest radiograph showed adequate re‐expansion of the lungs (Figure 1B).

Upon further history taking, it was noted that this was his first episode of pneumothorax. Over the last 3 months, he had a persistent dry cough that was not bothersome and had mild dyspnoea on exertion when hurrying or walking at an incline. He had a past medical history of childhood asthma for which he was not currently on any inhalers and had not suffered an exacerbation since he was 6 years old. He was working as a chef and was an active smoker, smoking half a pack a day for the last 5 years.

Routine blood tests were normal, including a normal full blood count with no polycythemia.

Given that bilateral pneumothorax was not a common presentation of primary spontaneous pneumothorax, an underlying lung disease was suspected. Detailed examination of the chest radiographs taken before and after chest tube insertion revealed bilateral airspace lucencies (Figure 1C,D), suspicious of underlying cystic lung disease.

**FIGURE 2:**
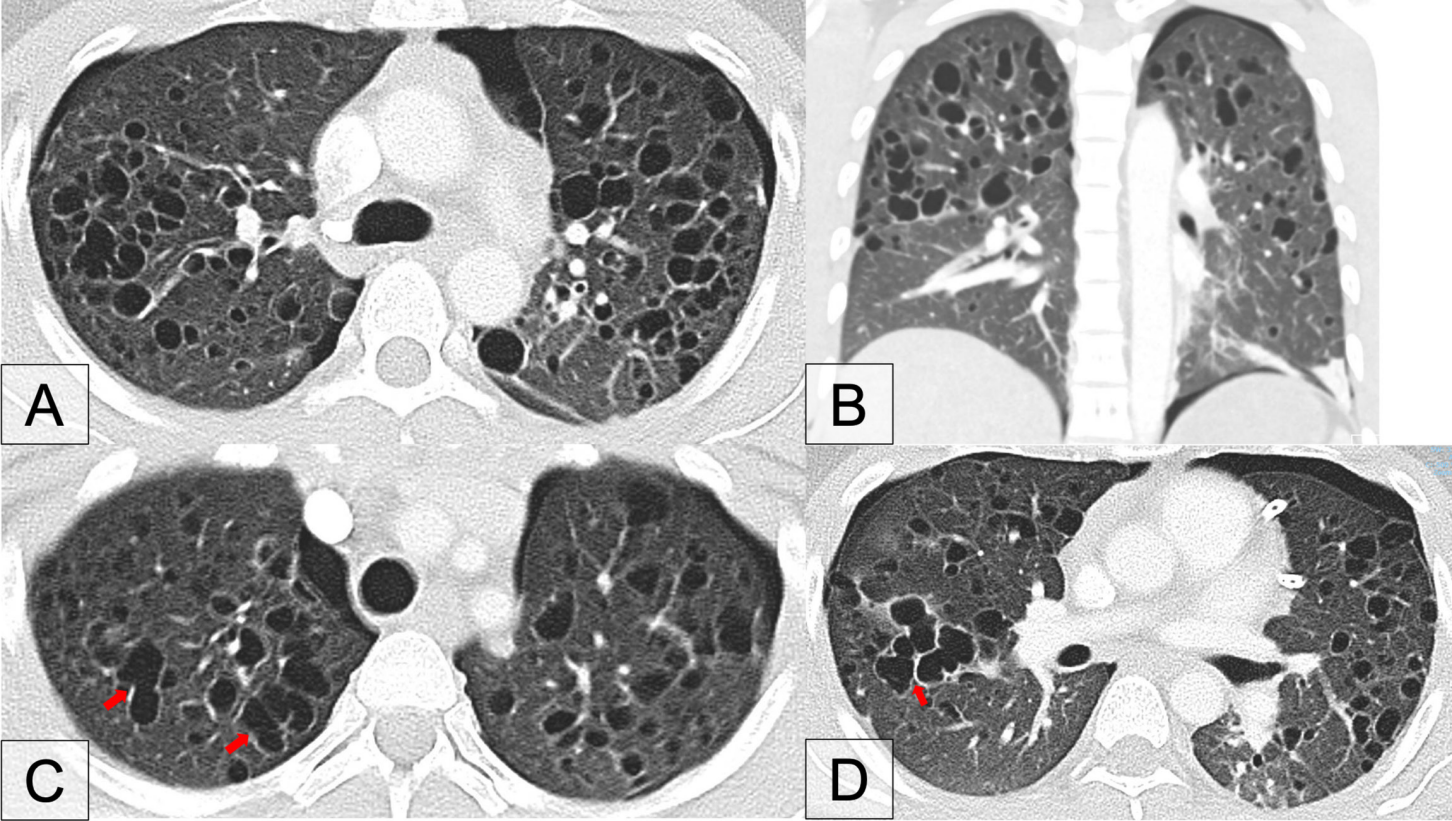
A: Diffused bilateral multiple thin‐walled cysts of varying sizes with tiny peripheral nodules and normal intervening parenchyma. B: Relative sparing of lower lobes. C: Bilobed appearance (arrows). D: Cloverleaf appearance (arrow).

A computed tomography (CT) scan of the chest was performed which revealed diffused bilateral scattered thin‐walled cysts of varying sizes with relative sparing of the lower lobes; tiny peripheral nodules; and normal intervening lung parenchyma. There were no enlarged intrathoracic lymph nodes or ground glass opacities. (Figure 2A,B) More detailed examination of the images revealed relative sparing of the lower lobes, tiny peripheral nodules, and confluence of multiple cysts that resulted in bizarre‐shaped cysts such as bilobed (Figure 2C) and cloverleaf (Figure 2D) appearances.

The patient was counselled for definitive surgical management of his bilateral pneumothorax and surgical biopsy for underlying cystic lung disease. He subsequently underwent bilateral video‐assisted thoracoscopic surgery (VATS) bullectomy, bilateral mechanical pleurodesis, and surgical lung biopsy. Mechanical pleurodesis is the process by which pleural adhesions are induced by physical abrasion of the pleural surfaces during surgery. Bilateral surgical pleurodesis was chosen in this case as the patient was already planned for surgical lung biopsy for evaluation of cystic lung disease and it was important to prevent further recurrence of pneumothorax given the severity of the patient's presentation, i.e., bilateral, symptomatic and hypoxemic.

The operation was carried out in a single two‐hour session with sequential single lung ventilation and VATS carried out on the contralateral deflated lung. Sequential single lung ventilation involves one‐lung ventilation to facilitate surgery on the contralateral lung, subsequently switching ventilation to the successfully operated lung to complete bilateral thoracic surgery.

The entire process was streamlined and efficient. Bilateral 14 Fr chest tubes were inserted upon admission to the ED after chest radiograph confirmed bilateral pneumothorax. CT imaging was done just 2 days after the patient's presentation to the ED. Surgery was then planned and performed 5 days after CT imaging, during which the bilateral chest tubes were connected to underwater seal without suction and there was no persistent bubbling to suggest active airleak.

**FIGURE 3:**
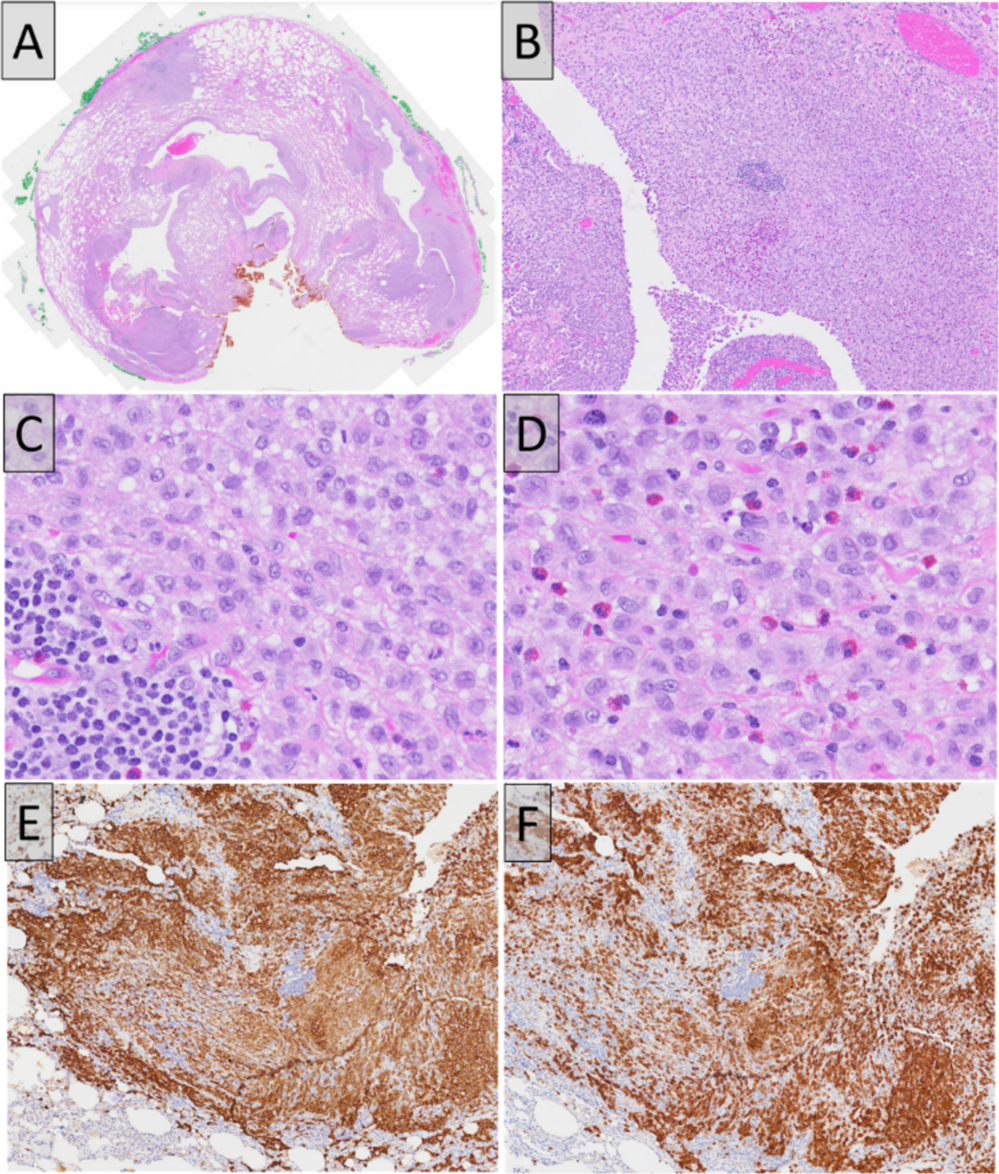
A: Low magnification of the lung parenchyma showed multiple cystic spaces. B: Higher magnification of the cyst spaces lined by sheets of Langerhans cells. C: The Langerhans cells showed grooved nuclei and a moderate to abundant amount of amphophilic cytoplasm. D: Numerous eosinophils are admixed with the Langerhans cells. The Langerhans cells were positive for E: CD1a and F: Langerin.

Gross pathological examination revealed scattered cystic spaces ranging from 0.1 cm to 0.7 cm and multiple pleural blebs ranging from 0.5 cm to 2 cm. Microscopic examination showed multiple cystic spaces (Figure 3A,B) lined by clusters and sheets of Langerhans cells showing grooved nuclei and a moderate to abundant amount of amphophilic cytoplasm (Figure 3C), admixed with numerous eosinophils (Figure 3D). On immunohistochemistry, the Langerhans cells were positive for CD1a (Figure 3E) and Langerin (Figure 3F), establishing the diagnosis of Pulmonary Langerhans cell histiocytosis (PLCH).

Comprehensive history and clinical examination did not reveal suggestion of other organ involvement or any skin lesions or syndromic features, confirming isolated Pulmonary Langerhans cell histiocytosis.

He recovered well post‐surgery and was advised on smoking cessation. He was offered counselling and pharmacological support but declined. Initial pulmonary function testing done 6 months post‐surgery showed a restrictive pattern. On subsequent follow ups, he has remained smoke‐free; his symptoms of dry cough and exertional dyspnoea have resolved; and there was improvement in his lung function (Table 1).

**TABLE 1:** Serial pulmonary function test post‐surgery.

Months post‐surgery	FEV_1_, L (% predicted)	FVC, L (% predicted)	FEV_1_/FVC, %	TLC, L (% predicted)	DLCO, mM/min/kPa (% predicted)
6 months	2.77 (74)	3.13 (73)	88.3	4.22 (68)	6.84 (68)
12 months	2.92 (77)	3.37 (77)	87	4.59 (73)	7.11 (70)

Abbreviations: DLCO, diffusing capacity of the lungs for carbon monoxide; FEV_1_, forced expiratory volume in 1 second; FVC, forced vital capacity; TLC, total lung capacity.

## 3 Discussion

The patient's young age and first presentation of bilateral pneumothorax should alert clinicians to the possibility of an underlying lung condition causing bilateral secondary spontaneous pneumothorax.

Differential diagnoses for the patient's presentation and underlying cystic changes in the lungs noted on the CT scan include PLCH, Lymphangioleiomyomatosis (LAM) and Birt–Hogg–Dubé (BHD) syndrome. The distinguishing clinical and radiological features of the differential diagnoses are described in Table 2 [2, 3].

**TABLE 2:** Distinguishing clinical and radiological features of PLCH, LAM, and BHD syndrome.

Differential diagnosis	Clinical features	Radiological features
Pulmonary Langerhans cell histiocytosis	Seen almost exclusively in smokers or ex‐smokers 20–40 years of age Common symptoms are cough, dyspnoea, fatigue, weight loss and spontaneous pneumothorax	Multiple, thin‐walled cysts predominantly upper and middle lobes Variable in size Bizarre shaped cysts Spares costophrenic angles Micronodules May have intervening architectural distortion
Lymphangioleiomyomatosis	May be associated with tuberous sclerosis or occurs sporadically Sporadic LAM is seen almost exclusively in women of childbearing age Common symptoms are slowly progressive dyspnoea, spontaneous pneumothorax, haemoptysis, chyloxthorax, or chylous ascites	Thin‐walled cysts surrounded by normal parenchyma Renal angiomyolipomas may also be seen
Birt–Hogg–Dubé syndrome	Autosomal dominant syndrome May present with pneumothorax Associated with fibrofolliculomas of the face, neck and upper trunk and renal tumours	Multiple, thin‐walled cysts predominantly seen in peripheral lung zones at lung bases and along the mediastinum Normal intervening lung parenchyma

A history of smoking coupled with the presence of multiple confluent bizarre‐shaped cysts that spare the lower lobes with normal intervening lung suggests a probable diagnosis of PLCH.

PLCH is a rare cystic lung disease that occurs almost exclusively in young smokers or ex‐smokers aged 20 to 40. Aetiology is unknown and is characterised by focal Langerhans cells granulomas infiltrating and destroying distal bronchioles. PLCH is one of the manifestations of Langerhans cell histiocytosis but mostly occurs in isolation [4].

Definite diagnosis of PLCH is made by histological confirmation of peribronchiolar infiltration by Langerhans and inflammatory cells and formation of granulomas. Without pathology, a diagnosis can be made with a combination of compatible clinical and radiological features, supported by bronchoscopic evaluation [5]. (Table 3).

**TABLE 3:** Diagnosis of pulmonary Langerhans cell histiocytosis.

Definite [4]	Histopathological confirmation of peribronchial inflammatory lesions containing an admixture of Langerhans cells, eosinophils, lymphocytes, and neutrophils. Langerhans‐like cells express CD1a, langerin (CD207), and S100.	
Without pathology	Clinical [4]	Typical age range 20–40 years Current or former cigarette smoker History of pneumothorax Common symptoms are cough, dyspnoea, constitutional symptoms May have polydipsia and polyuria from diabetes insipidus, pain from bone lesions or skin rashes
	Radiological [4]	Centrilobular or peribronchiolar nodules and cavities Thin‐walled cysts of varying sizes which can coalesce to form thick, irregular walled, bizarre‐shaped cysts Distribution in upper and middle zones with sparing of lung bases and costophrenic angles
	Bronchoalveolar lavage (BAL) [4, 5]	Presence of BAL fluid > 5% CD1a‐positive cells can be supportive to the diagnosis of PLCH in the absence of pathological confirmation.

For our patient, low magnification examination of his lung parenchyma confirmed the presence of multiple cystic spaces seen on CT scan. Upon higher magnification, the cyst spaces were lined with sheets of Langerhans cells admixed with eosinophils. On immunohistochemistry, the Langerhans cells were positive for CD1a and CD207 (Langerin), markers that are specific for LCH. This was pathological confirmation for PLCH.

Remarkably, our patient improved clinically after just smoking cessation. The effects of smoking cessation on PLCH varies, with some case reports describing improvements of pulmonary [6, 7, 8, 9] and extra‐pulmonary involvement [10] and a study showing no significant associations [11]. There is relative paucity of data regarding the direct effects of smoking cessation on PLCH progression and outcomes, but ongoing smoking has been identified as a risk factor for lung function decline [12]. In a recent study, the prognosis of PLCH at 10 years is estimated at 93%, and older age and poorer lung function were associated with higher risk of mortality [13]. Other factors associated with worse outcomes include multi‐organ involvement, extensive cysts and honeycombing and pulmonary hypertension [14, 15].

As such, smoking cessation remains the mainstay of treatment for PLCH due to its strong association and greater risk of lung function decline. Glucocorticoid therapy can be considered for patients who are unresponsive or have progressive disease despite smoking cessation.

## Author Contributions

I thank Drs Tzy Harn Chua and Chun Yuen Chow for providing the relevant pathological images and their invaluable input on the histopathological descriptions. I thank Dr. Yi Hern Tan for his guidance and help in writing this case report.

## Consent

The authors declare that written informed consent was obtained for the publication of this manuscript and accompanying images and attest that the form used to obtain consent from the patient complies with the Journal requirements as outlined in the author guidelines.

## Conflicts of Interest

The authors declare no conflicts of interest.

## Data Availability

The data that support the findings of this study are available on request from the corresponding author. The data are not publicly available due to privacy or ethical restrictions.
